# Prognostic impact of serum levels of EGFR and EGFR ligands in early-stage breast cancer

**DOI:** 10.1038/s41598-020-72944-1

**Published:** 2020-10-06

**Authors:** Ina Mathilde Kjær, Dorte Aalund Olsen, Ivan Brandslund, Troels Bechmann, Erik Hugger Jakobsen, Søren Bie Bogh, Jonna Skov Madsen

**Affiliations:** 1 Department of Biochemistry and Immunology, Lillebaelt Hospital, University Hospital of Southern Denmark, Vejle, Denmark; 2 Department of Regional Health Research, Faculty of Health Sciences, University of Southern Denmark, Odense, Denmark; 3 Department of Oncology, Lillebaelt Hospital, University Hospital of Southern Denmark, Vejle, Denmark; 4 OPEN, Open Patient Data Explorative Network, Department of Clinical Research, University of Southern Denmark, Odense, Denmark

**Keywords:** Breast cancer, Prognostic markers

## Abstract

Epidermal growth factor receptor (EGFR) and its ligands are involved in cancer pathogenesis. The emerging role of treatments co-targeting the EGFR system in breast cancer has increased the need to identify companion biomarkers. The aim of this study is to investigate whether pretreatment serum levels of EGFR and EGFR ligands in early-stage breast cancer patients might provide prognostic information as a stepping stone for further investigation. The study, which included 311 early-stage breast cancer patients, investigated associations between preoperative serum levels of EGFR and EGFR ligands (epidermal growth factor, heparin-binding epidermal growth factor (HBEGF), amphiregulin, transforming growth factor-α and betacellulin) and survival. Cutoffs were determined using Youden’s method, and overall survival (OS) and invasive disease-free survival (IDFS) were evaluated using Cox regression. Preoperative S-EGFR < 60.3 ng/mL was associated with shorter OS and IDFS in both univariate analyses and when adjusting for standard prognostic factors (p < 0.05). Preoperative S-HBEGF < 21.4 pg/mL was associated with shorter OS in both univariate and multivariate analyses, whereas association with shorter IDFS could only be demonstrated in the univariate analysis. In conclusion, our study demonstrated shorter survival in early-stage breast cancer patients who had low pretreatment levels of either S-EGFR or S-HBEGF.

## Introduction

The family of epidermal growth factor receptors, which consists of four related transmembrane receptors (EGFR, HER2, HER3 and HER4), is involved in cancer pathogenesis^1^. EGFR is activated upon by ligand binding, resulting in the activation of a complex intracellular pathway^1^. Among the ligands that activate EGFR are epidermal growth factor (EGF), heparin-binding epidermal growth factor (HBEGF), amphiregulin (AREG), transforming growth factor α (TGF-α) and betacellulin (BTC)^1^. EGFR-targeted therapies have well-established roles in the treatment of subgroups of lung cancer patients and colorectal cancer patients^2,3^. In breast cancer, however, most studies conducted so far could not identify benefits from applying EGFR targeted treatments and one of the hypotheses explaining the disappointing results is the heterogeneous nature of breast cancer and lack of valid predictive biomarkers to enable selection of patients who are most likely to respond to treatment^4–6^. Whereas the potential role of EGFR-targeted treatments in breast cancer is still undetermined, treatments targeting HER2 have radically improved outcomes for breast cancer patients with tumors overexpressing HER2^7,8^. Furthermore, recently, dual and pan-targeted EGFR inhibitors have also been implemented to treat breast cancer patients^9–11^. As more EGFR-targeted treatments are developed, there is an increasing need to identify companion biomarkers to predict treatment response and monitor treatment effect to improve patient outcomes. In colorectal and lung cancer, research indicates that blood levels of EGFR and EGFR ligands might serve as companion biomarkers for EGFR-targeted treatments^12–15^.

We conducted a systematic review to investigate whether EGFR and EGFR ligands might serve as prognostic or predictive blood-based biomarkers in breast cancer^16^. Several studies have investigated S-EGFR in the metastatic breast cancer setting, three of which report associations between low S-EGFR and shorter survival and reduced response to certain treatments^17–19^. EGFR ligands have been investigated to a much lesser extent, and several ligands have not been investigated at all^16^. In the systematic review, we identified only a single study that evaluated the prognostic value of S-EGFR in early-stage breast cancer patients, which included only 119 patients^20^, and we found no studies that evaluated the prognostic or predictive value of any of the EGFR ligands in early-stage breast cancer patients^16^. We recently repeated the literature search performed in 2017 when conducting our systematic review^16^ and found no additional studies in early-stage breast cancer patients. Thus, the prognostic and predictive significance of EGFR and EGFR ligands as blood-based biomarkers in early-stage breast cancer remains undetermined.

The primary aim of the present study was to investigate whether preoperative serum levels of EGFR and EGFR ligands were associated with overall survival and invasive disease-free survival in early-stage breast cancer patients.

## Methods

### Study design and patients

From 2004 to 2013, 383 newly diagnosed breast cancer patients gave written informed consent to participate in a prospective biomarker study at Lillebaelt Hospital, Vejle, Denmark. In the present study we conducted additional retrospective analyses of serum levels of EGFR and EGFR ligands. The inclusion criterion for the present study was primary diagnosed early-stage breast cancer, which could be surgically removed. Exclusion criteria included ducal carcinoma in situ (n = 4), primary advanced disease (n = 8) and neoadjuvant treatment (n = 5) (Table 1). Furthermore, patients with no available preoperative serum sample (n = 55) were excluded, leaving a total of 311 early-stage breast cancer patients to be included in the study (Table 1).

**Table 1:** REMARK profile^21^.

Biomarkers and covariates	Variables	Sample points	Cutoff
Biomarkers	S-EGFR S-EGF S-HBEGF S-AREG S-TGFα S-BTC	Baseline sample before primary surgery (n = 311) Postoperative sample 14 − 30 days after surgery (n = 113) Recurrence sample within 3 months before systemic metastatic recurrence (n = 14)	Cutoffs determined using Youden’s method for estimation of optimal cutoff
Covariates^a^	Age Type of surgery Pathology Adjuvant treatment		

Patients	n	Remarks	
Assessed for eligibility	383	Patients with newly diagnosed breast cancer at Lillebaelt Hospital, Vejle, Denmark, 2004 − 2013	
Excluded	72	Benign pathology (n = 4), primary advanced disease (n = 8), neoadjuvant chemotherapy (n = 5), missing preoperative serum sample (n = 55)	
Included	311		
**Outcome events**^**b**^			
Overall survival (OS)	78		
Invasive disease-free survival (IDFS)	108	First IDFS-event: Recurrence of breast cancer (n = 69), second primary non-breast cancer (n = 19), death (n = 20)	

^a^All covariates are presented in detail in Table 2.

^b^Study period ended April 24, 2019.

Patients underwent primary breast cancer surgery according to the guidelines of the Danish Breast Cancer Cooperative Group (DBCG) and subsequently received adjuvant treatment according to existing guidelines.

Histopathological data were obtained from the local pathology database at Lillebaelt Hospital, Denmark. Clinical information was obtained from local electronic medical records at Lillebaelt Hospital, Denmark. The study period ended on April 24, 2019.

The study was approved by the Regional Committees on Health Research Ethics for Southern Denmark (S-20170119) and the Danish Data Protection Agency (journal number 8/56003) and was conducted in accordance with the Helsinki Declaration.

The study adheres to the Reporting Recommendations for Tumor Marker Prognostic Studies (REMARK)^21^.

### Samples

Baseline and follow-up blood samples were collected using standard venipuncture procedure performed by skilled phlebotomists, centrifuged, aliquoted and stored at − 80 °C in a biobank at Lillebaelt Hospital, Vejle, Denmark.

Baseline serum samples were obtained 1–42 days (mean 7 days) prior to primary surgery for breast cancer. A postoperative sample was obtained between 14 and 30 days after primary surgery. Serum samples were subsequently obtained at standard clinical control visits. In the present study we included follow-up samples from patients who experienced metastatic recurrence. These samples were obtained within three months prior to a diagnosis of a systemic recurrence of breast cancer.

### Assays

Serum levels of EGFR (S-EGFR) and EGF (S-EGF) were measured using sandwich enzyme-linked immunosorbent assay (ELISA) testing. HBEGF (S-HBEGF), AREG (S-AREG), TGF-α (S-TGFα) and BTC (S-BTC) were analyzed using ultra-sensitive Single Molecule Array (Simoa) technology^22^. S-AREG, S-TGFα and S-BTC were analyzed using a three-plex assay^23^. All assays used in the present study have previously been described in detail^23,24^. During analysis, the serum samples from the individual patients were analyzed in the same run. Each run included at least three assay controls. The total coefficients of variation (CV%) were: S-EGFR 8–17%, S-EGF 8–12%, S-HBEGF 15–29%, S-AREG 12–21%, S-TGFα 8–14% and S-BTC 11–25%. The limits of detection were: S-EGFR 0.014 ng/mL, S-EGF 0.03 pg/mL, S-HBEGF 0.05 pg/mL, S-AREG 0.16 pg/mL, S-TGFα 0.2 pg/mL and S-BTC 0.2 pg/mL.

The estrogen receptor (ER) status and progesterone receptor (PR) status of the breast cancer tumor was determined using immunohistochemical staining (IHC) and, according to contemporary guidelines, the tumor was considered positive if ≥ 10% of nuclei were stained. The HER2 status of the tumor was determined using IHC and fluorescence in situ hybridization (FISH). The tumor was considered HER2-positive in cases with IHC3 + or IHC 2 + and FISH > 2, whereas the tumor was considered HER2-negative in cases with IHC 0, IHC 1 + , or IHC 2 + and FISH < 2.The ER and HER2 assays have been described in a previous study by Kjær et al^25^.

The laboratory technicians performing analysis of the serum samples were blinded for all clinical information including outcome for the patients.

### Clinical end points

Outcome measures were defined according to the Proposal for Standardized Definitions for Efficacy End Points in Adjuvant Breast Cancer Trials: The STEEP system^26^. Invasive Disease-Free Survival (IDFS) was defined as the time from primary breast cancer surgery until the occurrence of one of the following events: invasive ipsilateral breast tumor recurrence; local/regional invasive recurrence; distant recurrence; invasive contralateral breast cancer; second primary invasive non-breast cancer (with the exception of squamous or basal cell skin cancers) or death by any cause. All in-situ cancer events, from both breast and non-breast sites, are excluded from the IDFS definition^26^. Overall Survival (OS) was defined as the time from primary breast cancer surgery to death, including death from breast cancer, any other cause or unknown cause^26^.

### Statistical methods

As the prognostic value of EGFR and EGFR ligands in breast cancer is investigated to a very limited extend^16^, the present study was conducted with an explorative approach and no specified effect size was expected.

First, an optimal cutoff for each biomarker was estimated using Youden’s method with logistic regressions^27^. The logistic regressions were conducted with 5-year overall survival as a dependent variable and baseline biomarker level as an independent variable. In the S-AREG, S-TGFα and S-BTC variables, but not in the S-EGFR, S-EGF and S-HBEGF variables, outliers were identified in the baseline samples of seven patients and removed. These outliers could be due to heterophilic antibodies, rheumatoid factors or non-specific binding that caused interference specifically in the three-plex Simoa assay. The distributions of data for S-AREG, S-TGFα and S-BTC showed highly skewed distributions; hence, logistic regressions were conducted using log-transformed independent variables. The cutoff for S-AREG, S-TGFα and S-BTC were then back-transformed to the original scale.

Second, univariate Cox regressions were performed for all biomarkers using Youden cutoffs, and for the standard prognostic covariates including age, type of surgery, pathology and adjuvant treatment. The two endpoints, OS and IDFS, were evaluated. We created Kaplan Meier curves depicting OS in the groups delineated by Youden cutoffs for S-EGFR and S-HBEGF. Multivariate Cox regressions were performed for each of the biomarkers using Youden cutoffs. The models were adjusted for all standard prognostic covariates. The two endpoints, OS and IDFS, were evaluated. The assumption of proportional hazards was investigated using Schoenfeld residuals.

Third, Cox regressions for OS and IDFS were performed for all six biomarkers in the subgroups defined as hormone receptor positive patients (ER-positive and/or PR-positive), HER2-positive patients and triple negative patients (hormone receptor negative and HER2-negative).

Stata IC 16.1 software package (StataCorp. 2019. Stata Statistical Software: Release 16. College Station, TX: StataCorp LLC) was used for the statistical analysis.

## Results

### Patient characteristics

A total of 311 women with early-stage breast cancer were included in the study. The clinical-pathological characteristics, including standard prognostic parameters, are presented in Table 2. The population of women with early-stage breast cancer included in the present study has previously been described in a study by Kjær et al^25^. As reported, the clinical-pathological characteristics of the study population are considered to be representative of patients with early-stage breast cancer^25^. The adjuvant therapy is presented in Table 2. A total of 269 patients (86.5%) received adjuvant radiotherapy, 146 patients (46.9%) received adjuvant chemotherapy, 220 patients (70.7%) received endocrine treatment and 38 patients (12.2%) received HER2-targeted treatment. Median and interquartile range of the preoperative serum levels of EGFR and EGFR ligands are presented in Table 2. Furthermore, the optimal cutoff for each biomarker, estimated using Youden’s method, is presented, including the number of patients with levels above and below these cutoffs.

**Table 2:** Characteristics of the population of early-stage breast cancer patients including histopathological information (previously published by Kjær et al.^25,33^) and adjuvant treatment. Median and interquartile range (IQR) of preoperative EGFR and EGFR ligand serum levels (previously published by Kjær et al.^25,33^) and EGFR and EGFR ligand cutoffs determined using Youden’s method for estimation of optimal cutoff are presented along with the number of patients with levels above and below the Youden cutoffs.

Early-stage breast cancer patients, n (%)		
N	311	
**Age**		
< 50 years	65 (20.9%)	
50–69 years	197 (63.3%)	
≥ 70 years	49 (15.8%)	
**Type of surgery**		
Lumpectomy	243 (78.1%)	
Mastectomy	68 (21.9%)	
**Tumor type**^a^		
Ductal	276 (88.7%)	
Lobular	15 (4.8%)	
Other	20 (6.4%)	
**Tumor grade**		
Grade I	71 (22.8%)	
Grade II	141 (45.3%)	
Grade III	78 (25.1%)	
Unknown	21 (6.8%)	
**Tumor size**		
T1 ≤ 20 mm	175 (56.3%)	
T2 > 20 ≤ 50 mm	132 (42.4%)	
T3 > 50 mm	4 (1.3%)	
**Nodal status**		
N0 0 nodes	155 (49.8%)	
N1 1–3 nodes	111 (35.7%)	
N2-3 ≥ 4 nodes	45 (14.5%)	
**HR status**^b^		
ER- and PR-/unknown	58 (18.6%)	
ER + and/or PR +	253 (81.4%)	
**ER status**		
Negative (< 10%)	58 (18.6%)	
Positive (10–100%)	253 (81.4%)	
**PR status**		
Negative (< 10%)	91 (29%)	
Positive (10–100%)	183 (59%)	
Unknown	37 (12%)	
**HER2 status**^c^		
Negative	248 (79.7%)	
Positive	49 (15.8%)	
Unknown	14 (4.5%)	
**Triple negative**		
Yes	33 (11%)	
No	227 (73%)	
Unknown	51 (16%)	
**Adjuvant chemotherapy**^d^		
No	165 (53.1%)	
EC and D	77 (24.8%)	
CEF or EC	58 (18.6%)	
DC	9 (2.9%)	
Other	2 (0.6%)	
**Adjuvant HER2-targeted treatment**		
No	273 (87.8%)	
Trastuzumab	24 (7.7%)	
Trastuzumab and TKI	11 (3.5%)	
TKI	3 (1.0%)	
**Adjuvant endocrine treatment**		
No	91 (29.3%)	
Tamoxifen	51 (16.4%)	
Tamoxifen and AI	100 (32.2%)	
AI	69 (22.2%)	
**Adjuvant radiotherapy**		
No	42 (13.5%)	
Yes	269 (86.5%)	

Preoperative biomarker level	Median (IQR)	Youden cutoff (n < cutoff, n ≥ cutoff, missing/outlier)
S-EGFR, ng/mL	68 (62, 78)	60.3 *(62, 249, 0)*
S-EGF, pg/mL	490 (314, 768)	296 *(73, 238, 0)*
S-HBEGF, pg/mL	30.1 (23.7, 39.3)	21.4 *(56, 253, 2)*
S-AREG, pg/mL	2.6 (1.7, 5.2)	5.3 *(234, 70, 7)*
S-TGFα, pg/mL	7.8 (4.8, 12)	8.2 *(164, 140, 7)*
S-BTC, pg/mL	8.3 (4.3, 18.6)	9.6 *(165, 139, 7)*

Four patients (1.3%) had bilateral synchronous breast cancer. In these cases, the pathological data represent the tumor with the severest prognostics.

^a^Tumor type was considered ductal in cases where other histological types were detected in the tumor in addition to ducal carcinoma.

^b^HR status: Hormone receptor status in breast cancer tumor determined by estrogen receptor status and progesterone receptor status. Positive: ER + and/or PR + . Negative: ER- and PR- (in two cases ER- and PR unknown).

^c^HER2 status: Status of human epidermal growth factor receptor 2 (HER2) in breast cancer tumor evaluated by immunohistochemistry (IHC) and fluorescence in situ hybridization (FISH). Positive: IHC 3 + or IHC 2 + and FISH > 2. Negative: IHC 0 or IHC 1 + or IHC 2 + and FISH < 2.

^d^EC + D: Epirubicin, cyclophosphamide and docetaxel; CEF: Cyclophosphamide, epirubicin and fluorouracil; EC: Epirubicin and cyclophosphamide; DC: Docetaxel and cyclophosphamide; Other: cyclophosphamide, methotrexate and fluorouracil (CMF) or taxol.

ER status: Estrogen receptor status; PR status: Progesterone receptor status; TKI: Tyrosine kinase inhibitor; AI: Aromatase inhibitor.

### Overall survival

The median follow-up time for OS was 11.0 years, and a total of 78 of the 311 patients included in the study died at some point during the study period. The five-year OS rate was 90%.

**Figure 1:**
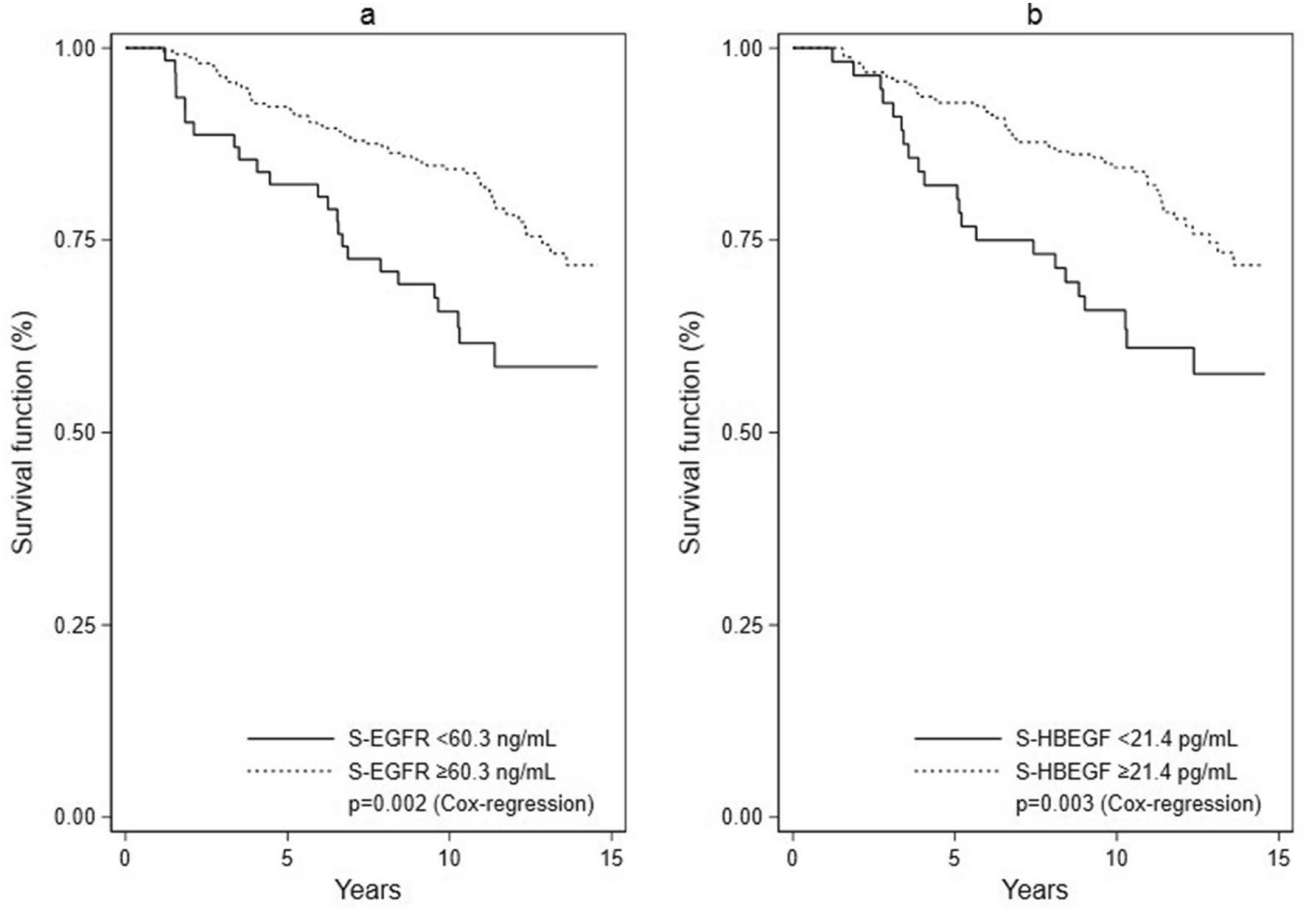
Kaplan–Meier curves showing overall survival in 311 early-stage breast cancer patients. Patients were divided into groups based on preoperative level of S-EGFR (**a**) or S-HBEGF (**b**). Cutoffs were determined using Youden’s method for estimation of optimal cutoff. P-values were determined using Cox-regressions.

S-EGFR: Univariate Cox regression showed that baseline S-EGFR below 60.3 ng/mL (Youden-estimated cutoff) was associated with shorter OS (p = 0.002) (Table 3). Kaplan–Meier curves are presented in Fig. 1. Moreover, in multivariate Cox regression (adjusted for all covariates), baseline S-EGFR < 60.3 ng/mL was associated with shorter survival (p = 0.01). Cox analyses were conducted in subgroups defined as hormone receptor positive patients, HER2-positive patients and triple negative patients (Table 4a). Hormone receptor positive patients with S-EGFR < 60.3 ng/mL showed a significantly shorter OS (p = 0.01) than patients with S-EGFR ≥ 60.3 ng/mL. However, when adjusting for HER2 status, the association did not reach statistical significance (p = 0.06). HER2-positive patients with S-EGFR < 60.3 ng/mL showed a significantly shorter OS (p = 0.002) in both univariate analysis and when adjusting for hormone receptor status (p = 0.007). In the triple negative subgroup, no association between OS and S-EGFR was observed.

**Table 3:** Univariate and multivariate Cox regression analysis of overall survival (OS) and invasive disease-free survival (IDFS) in early-stage breast cancer patients in relation to EGFR and EGFR ligand serum levels.

	OS				IDFS			
	Univariate		Multivariate		Univariate		Multivariate	
	HR (95% CI)	P	HR (95% CI)	P	HR (95% CI)	P	HR (95% CI)	P
**S-EGFR**^a^								
< 60.3 ng/mL	–		–		–		–	
≥ 60.3 ng/mL	0.5 (0.3–0.8)	**0.002**	0.5 (0.3–0.9)	**0.01**	0.5 (0.4–0.8)	**0.004**	0.6 (0.4–0.9)	**0.015**
**S-EGF**^a^								
< 296 pg/mL	–		–		–		–	
≥ 296 pg/mL	0.7 (0.4–1.1)	0.1	0.8 (0.5–1.4)	0.4	0.7 (0.5–1.1)	0.09	0.9 (0.5–1.3)	0.5
**S-HBEGF**^a^								
< 21.4 pg/mL	–		–		–		–	
≥ 21.4 pg/mL	0.5 (0.3–0.8)	**0.003**	0.5 (0.3–0.9)	**0.01**	0.6 (0.4–0.9)	**0.008**	0.6 (0.4–1.0)	0.05
**S-AREG**^a^								
< 5.3 pg/mL	–		–		–		–	
≥ 5.3 pg/mL	1.2 (0.7–2.0)	0.5	1.3 (0.8–2.3)	0.3	1.0 (0.7–1.6)	0.9	1.0 (0.6–1.6)	0.9
**S-TGFα**^a^								
< 8.2 pg/mL	–		–		–		–	
≥ 8.2 pg/mL	0.8 (0.5–1.2)	0.3	0.8 (0.5–1.3)	0.4	0.8 (0.5–1.2)	0.2	0.8 (0.5–1.2)	0.2
**S-BTC**^a^								
< 9.6 pg/mL	–		–		–		–	
≥ 9.6 pg/mL	0.7 (0.5–1.2)	0.2	0.8 (0.5–1.3)	0.4	0.7 (0.5–1.1)	0.1	0.7 (0.5–1.1)	0.2
**Age (years)**								
< 50	–				–			
50–69	2.0 (0.9–4.2)	0.08			1.6 (0.9–2.7)	0.1		
≥ 70	6.5 (3.0–14)	** < 0.001**			3.4 (1.8–6.3)	** < 0.001**		
**Surgery**								
Lumpectomy	–				–			
Mastectomy	1.6 (1.0–2.7)	**0.04**			1.4 (0.9–2.2)	0.1		
**Tumor type**^b^								
Ductal	–				–			
Lobular	1.0 (0.4–2.7)	1.0			0.9 (0.4–2.1)	0.7		
Other	0.7 (0.3–2.0)	0.6			0.5 (0.2–1.3)	0.2		
**Grade**								
I, II, unknown	–				–			
III	2.2 (1.4–3.5)	**0.001**			1.9 (1.2–2.8)	**0.002**		
**Tumor type**								
≤ 20 mm	–				–			
> 20 mm	2.9 (1.8–4.6)	** < 0.001**			2.1 (1.4–3.1)	** < 0.001**		
**Nodal status**								
Negative	–				–			
Positive	1.1 (0.7–1.7)	0.7			1.2 (0.8–1.7)	0.4		
**HR status**^**c**^								
ER- and PR-/unknown	–				–			
ER + and/or PR +	0.6 (0.4–1.0)	0.05			0.7 (0.4–1.1)	0.1		
**HER2 status**^d^								
Negative	–				–			
Positive	1.3 (0.7–2.3)	0.4			1.1 (0.7–1.9)	0.6		
**Chemotherapy**								
No	–				–			
Yes	0.5 (0.3–0.8)	**0.002**			0.5 (0.4–0.8)	**0.003**		
**Endocrine treatment**								
No	–				–			
Yes	0.8 (0.5–1.4)	0.5			0.8 (0.5–1.2)	0.3		
**HER2-targeted treatment**								
No	–				–			
Yes	0.9 (0.4–1.8)	0.7			0.7 (0.4–1.4)	0.4		
**Radiotherapy**								
No	–				–			
Yes	0.6 (0.3–1.0)	0.05			0.7 (0.4–1.1)	0.1		

The multivariate Cox regressions were adjusted for all covariates (age, type of surgery, pathology, adjuvant treatment).

^a^Cutoffs determined using Youden’s methods for estimation of optimal cutoff.

^b^Tumor type was considered ductal in cases where other histological types were detected in the tumor in addition to ducal carcinoma.

^c^HR status: Hormone receptor status in breast cancer tumor determined by estrogen receptor status and progesterone receptor status (immunohistochemistry (IHC)). Positive: ER + and/or PR + . Negative: ER- and PR- (in two cases ER- and PR unknown).

^d^HER2 status: Status of human epidermal growth factor receptor 2 (HER2) in breast cancer tumor evaluated by IHC and fluorescence in situ hybridization (FISH). Positive: IHC 3 + or IHC 2 + and FISH > 2. Negative: IHC 0 or IHC 1 + or IHC 2 + and FISH < 2. HR: Hazard ratio; 95% CI: 95% confidence intervals.

**Table 4:** EGFR and EGFR ligands as prognostic factors in subgroups of early-stage breast cancer patients in relation to overall survival (a) and invasive disease-free survival (b).

a						
	Overall survival					
	HR + Patients: n = 253 Events: n = 58		HER2 + Patients: n = 49 Events: n = 14		Triple negative Patients: n = 33 Events: n = 10	
	HR (95% CI)	p	HR (95% CI)	p	HR (95% CI)	p
**S-EGFR**						
< 60.3 ng/mL	–		–		–	
≥ 60.3 ng/mL	0.5 (0.3–0.9)	**0.01**	0.2 (0.06–0.5)	**0.002**	1.1 (0.2–5.2)	0.9
Adjusted*	0.5 (0.3–1.0)	0.06	0.2 (0.07–0.7)	**0.007**	–	
**S-EGF**						
< 296 pg/mL	–		–		–	
≥ 296 pg/mL	0.5 (0.3–0.9)	**0.02**	2.2 (0.5–9.9)	0.3	0.7 (0.2–2.6)	0.7
Adjusted*	0.5 (0.3–1.0)	**0.04**	2.1 (0.5–9.5)	0.3	–	
**S-HBEGF**						
< 21.4 pg/mL	–		–		–	
≥ 21.4 pg/mL	0.5 (0.3–0.9)	**0.02**	0.5 (0.1–1.9)	0.3	0.3 (0.09–1.1)	0.07
Adjusted*	0.5 (0.3–0.8)	**0.01**	0.5 (0.1–1.9)	0.3	–	
**S-AREG**						
< 5.3 pg/mL	–		–		–	
≥ 5.3 pg/mL	1.1 (0.6–2.0)	0.8	3.6 (1.3–10.3)	**0.02**	1.5 (0.3–7.0)	0.6
Adjusted*	1.2 (0.6–2.2)	0.6	6.1 (2.0–18.8)	**0.001**	–	
**S-TGFα**						
< 8.2 pg/mL	–		–		–	
≥ 8.2 pg/mL	0.8 (0.5–1.4)	0.4	0.6 (0.2–1.9)	0.4	0.7 (0.2–2.7)	0.6
Adjusted*	0.8 (0.5–1.4)	0.5	0.7 (0.2–2.0)	0.5	–	
**S-BTC**						
< 9.6 pg/mL	–		–		–	
≥ 9.6 pg/mL	0.8 (0.4–1.3)	0.3	0.8 (0.3–2.2)	0.6	0.8 (0.2–2.9)	0.7
Adjusted*	0.8 (0.4–1.3)	0.4	0.8 (0.3–2.4)	0.7	–	

b						
	Invasive disease free survival					
	HR +		HER2 +		Triple negative	
	Patients: n = 253		Patients: n = 49		Patients: n = 33	
	Events: n = 84		Events: n = 18		Events: n = 12	
	HR (95% CI)	p	HR (95% CI)	p	HR (95% CI)	p
**S-EGFR**						
< 60.3 ng/mL	–		–		–	
≥ 60.3 ng/mL	0.6 (0.3–0.9)	**0.02**	0.3 (0.1–0.8)	**0.02**	0.9 (0.2–3.3)	0.9
Adjusted*	0.6 (0.4–1.0)	0.06	0.3 (0.1–1.0)	0.06	–	
**S-EGF**						
< 296 pg/mL	–		–		–	
≥ 296 pg/mL	0.6 (0.4–1.0)	0.07	0.9 (0.3–2.6)	0.9	0.7 (0.2–2.2)	0.6
Adjusted*	0.6 (0.4–1.0)	0.07	0.9 (0.3–2.5)	0.8	–	
**S-HBEGF**						
< 21.4 pg/mL	–		–		–	
≥ 21.4 pg/mL	0.6 (0.3–0.9)	**0.02**	0.8 (0.2–2.8)	0.7	0.4 (0.1–1.5)	0.2
Adjusted*	0.5 (0.3–0.9)	**0.01**	0.8 (0.2–2.8)	0.7	–	
**S-AREG**						
< 5.3 pg/mL	–		–		–	
≥ 5.3 pg/mL	0.9 (0.5–1.5)	0.6	2.7 (1.0–7.1)	**0.046**	1.7 (0.5–6.5)	0.4
Adjusted*	0.9 (0.6–1.6)	0.8	4.0 (1.4–11.0)	**0.008**	–	
**S-TGFα**						
< 8.2 pg/mL	–		–		–	
≥ 8.2 pg/mL	0.9 (0.5–1.3)	0.5	0.6 (0.2–1.5)	0.3	0.8 (0.2–2.5)	0.7
Adjusted*	0.8 (0.5–1.3)	0.4	0.6 (0.2–1.6)	0.3	–	
**S-BTC**						
< 9.6 pg/mL	–		–		–	
≥ 9.6 pg/mL	0.8 (0.5–1.2)	0.2	0.5 (0.2–1.4)	0.2	0.9 (0.3–3.0)	0.9
Adjusted*	0.7 (0.5–1.1)	0.1	0.5 (0.2–1.4)	0.2	–	

HR: Hazard ratio; HR + : Hormone receptor positive (estrogen receptor positive and/or progesterone receptor positive); 95% CI: 95% conficence interval; HER2 + : Human epidermal growth factor receptor 2 (HER2) positive evaluated by immunohistochemistry (IHC) and fluorescence in situ hybridization (FISH) (IHC 3 + or IHC 2 + and FISH > 2)

Cox-regressions conducted in subgroups of early-stage breast cancer patients including HR + patients, HER2-positive patients and triple negative patients, respectively. Cutoffs determined using Youden’s method for estimation of optimal cutoff applied to the entire population of early-stage breast cancer patients.

*In the category of HR + patients the adjusted analysis is adjusted for HER2 status. In the category of HER2 + patients the adjusted analysis is adjusted for hormone receptor status.

S-HBEGF: Baseline S-HBEGF < 21.4 pg/mL was associated with shorter OS in univariate Cox regression (p = 0.003), and this association persisted after adjusting for all covariates (p = 0.01). Kaplan–Meier curves are presented in Fig. 1. Hormone receptor positive patients with S-HBEGF < 21.4 pg/mL were found to have a significantly shorter OS both when performing univariate analysis (p = 0.02) and when adjusting for HER2 status (p = 0.01). No associations were found in the subgroup of HER2-positive patients or triple negative patients (Table 4a).

S-EGF, S-BTC, S-AREG and S-TGFα: No associations between preoperative serum levels of these biomarkers and OS were found in either univariate or multivariate Cox regression in the entire study population (Table 3). In the hormone receptor positive subgroup S-EGF < 296 pg/mL was associated with shorter OS both in univariate analysis (p = 0.02) and when adjusting for HER2 status (p = 0.04). In the HER2-positive subgroup S-AREG ≥ 5.3 pg/mL was associated with a significantly shorter OS both in univariate analysis (p = 0.02) and when adjusting for hormone receptor status (p = 0.001) (Table 4a).

### Invasive disease-free survival

The median follow-up time for IDFS was 10.6 years. During the follow-up period, 108 patients had one or more IFDS events (69 patients had recurrence of breast cancer as a first event, 19 patients had a second primary non-breast cancer as a first event and for 20 patients death was the first event). Five-year IDFS rate for the study population was 82%.

S-EGFR: Univariate Cox regression showed that baseline S-EGFR < 60.3 ng/mL was associated with shorter IDFS (p = 0.004) (Table 3). This association remained significant after applying a multivariate Cox regression (adjusted for all covariates) (p = 0.015). Hormone receptor positive patients with S-EGFR < 60.3 ng/mL showed a significantly shorter IDFS (p = 0.02) than patients with S-EGFR ≥ 60.3 ng/mL; however, when adjusting for HER2 status, the association did not reach statistical significance (p = 0.06). HER2-positive patients with S-EGFR < 60.3 ng/mL showed a significantly shorter IDFS (p = 0.02) in univariate analysis, but not when adjusting for hormone receptor status (p = 0.06). (Table 4b).

S-HBEGF: Baseline S-HBEGF < 21.4 pg/mL was associated with shorter IDFS in univariate Cox regression (p = 0.008) (Table 3). However, when adjusting for all covariates the association did not reach statistical significance (p = 0.05). Hormone receptor positive patients with S-HBEGF < 21.4 pg/mL were found to have significantly shorter IDFS both when performing univariate analysis (p = 0.02) and when adjusting for HER2 status (p = 0.01). No associations were found in the subgroup of HER2-positive patients (Table 4b).

S-EGF, S-BTC, S-AREG and S-TGFα: No associations between preoperative serum levels of these biomarkers and IDFS were found in either univariate or multivariate Cox regression in the entire study population (Table 3). In the HER2-positive subgroup S-AREG ≥ 5.3 pg/mL was associated with a significantly shorter IDFS both in univariate analysis (p = 0.046) and when adjusting for hormone receptor status (p = 0.008) (Table 4b).

### Pre- to postoperative changes of EGFR and EGFR ligands in serum

To evaluate whether pre- to postoperative changes in serum levels of EGFR and EGFR ligands might provide prognostic information in early-stage breast cancer patients, we conducted plots depicting the pre- to postoperative changes of each biomarker for the individual patients. The postoperative serum sample was obtained 14–30 days after primary breast cancer surgery and was available from 113 patients. Spaghetti plots depicting pre- to postoperative changes in patients alive and dead after five years showed no tendencies in the pre- to postoperative changes of any of the biomarkers and no relation to 5-year overall survival (Online Appendix 1). Moreover, waterfall plots illustrating pre- to postoperative delta-values showed no tendencies towards associations with 5-year overall survival (Online Appendix 2).

### Changes in serum levels of EGFR and EGFR ligands before systemic recurrence of breast cancer

To evaluate the potential of EGFR and EGFR ligands as predictive biomarkers in relation to earlier detection of metastatic recurrence of breast cancer, we conducted plots depicting the changes in serum levels of each biomarker before systemic recurrence of breast cancer. The recurrence serum sample was obtained within three months before systemic recurrence of breast cancer and was available from 14 individual patients. For each biomarker the preoperative level, postoperative level and level at time of systemic recurrence of breast cancer were depicted for these 14 patients (Online Appendix 3). The changes in biomarker levels during the course of disease varied between the individual patients and showed no distinct patterns, nor were distinct changes at time of recurrence observed.

## Discussion

The present study investigated the prognostic value of EGFR and EGFR ligands in the serum of 311 patients with early-stage breast cancer and demonstrated significantly shorter survival in patients with low pretreatment levels of either S-EGFR or S-HBEGF.

S-EGFR below the defined cut-off at 60.3 ng/mL was associated with shorter OS and IDFS in both univariate and multivariate analysis in the entire population. Subgroup analysis showed, that in the hormone receptor positive subgroup, patients with S-EGFR < 60.3 ng/mL had shorter OS and IDFS; however, when adjusting for HER2 status, the associations did not reach statistical significance. Similar analysis in the HER2-positive subgroup also showed shorter OS and IFDS in patients with low S-EGFR and here the association remained significant for OS after adjustment for hormone receptor status. Only one study has previously investigated the prognostic value of S-EGFR in early-stage breast cancer patients and found no association between preoperative S-EGFR and disease-free survival; however, the study included only 119 patients^20^. Furthermore, no subgroup analysis was conducted in the study^20^. Several studies have investigated the prognostic significance of S-EGFR in metastatic breast cancer patients^16,28^. Overall, the results of these studies indicate associations between low levels of S-EGFR and shorter survival^17–19,28^. Though some studies found no significant associations, no studies reported opposing results^16^. Few of the studies have investigated the prognostic significance of S-EGFR in subgroups: In relation to the hormone receptor positive subgroup two studies reported shorter survival in patients with low baseline S-EGFR^17,19^, which is in accordance to the findings of the present study. One other study found no correlation^29^. In relation to the HER2-positive subgroup, two previous studies found no association between S-EGFR and survival^10,30^. In conclusion, the results of the present study indicate shorter survival in patients with low S-EGFR not only in metastatic breast cancer as shown in previous studies, but also in early-stage breast cancer. Several potential mechanisms explaining the associations between low S-EGFR and shorter survival have been proposed and were discussed thoroughly in a study by Banys–Paluchowski^28^. However, current evidence is inconclusive, and further investigations into these mechanisms are recommended.

Preoperative S-HBEGF < 21.4 pg/mL was associated with shorter OS in both univariate and multivariate Cox regression, whereas association with shorter IDFS could only be demonstrated in the univariate analysis. No associations with OS or IFDS were found in the subgroup of HER2-positive patients, whereas hormone receptor positive patients with S-HBEGF < 21.4 pg/mL were found to have significantly shorter OS and IDFS in both univariate data analysis and when adjusting for HER2 status. No previous studies have investigated the prognostic value of S-HBEGF in breast cancer.

Regarding the remaining EGFR-ligands, we found no associations between preoperative level of either S-EGF, S-BTC, S-AREG or S-TGFα and OS or IDFS in the entire study population. However, when performing subgroup analysis, results showed that S-AREG ≥ 5.3 pg/mL was associated with shorter OS and IDFS in HER2-positive patients, both in univariate analysis and after adjusting for hormone receptor status. In addition, in the subgroup of hormone receptor positive patients S-EGF < 296 pg/ml was associated to shorter OS in both univariate analysis and when adjusting for HER2 status. Previous research investigating EGFR ligands in breast cancer are sparse and included solely HER2-positive patients^16,31^. Only S-EGF, S-TGFα and S-AREG have previously been investigated, whereas no investigation of S-BTC or S-HBEGF have been performed, to our best knowledge^16^. Furthermore, the study populations included advanced breast cancer patients and evaluated mainly the biomarkers in relation to treatment response^16,31^. Thus, to our best knowledge, this study is the first to evaluate the prognostic value of serum levels of EGFR ligands in early-stage breast cancer patients and across subgroups.

Pre-to postoperative changes in EGFR or EGFR ligand levels in serum showed no tendencies towards associations with 5-year overall survival (Online Appendices 1 and 2). Moreover, changes in EGFR and EGFR ligands during the course of disease varied between the individual patients and showed no distinct patterns or distinct changes at the time of systemic recurrence (Online Appendix 3). However, due to the low number of patients in these additional investigations, these findings should be interpreted with caution.

The present study has some limitations. First, breast cancer is known to be a heterogeneous disease with regard to hormone receptor status and HER2 status. In the present study distinct subgroups as defined by hormone receptor positive patients, HER2-positive patients and triple negative patients were too small to apply a complete multivariate analysis and the results of these subgroup analyses should be interpreted with utmost caution, in particular the triple negative subgroup. Thus, further studies investigating EGFR and EGFR ligands in large distinct subgroups of breast cancer patients are recommended. Second, because EGFR and EGFR ligands have been investigated as serological biomarkers to such a limited extent, reference methods and reference materials are unavailable. Whereas S-EGFR and S-EGF could be quantified using ELISA-testing, we developed highly sensitive immunoassays for S-HBEGF, S-AREG, S-TGFα and S-BTC on the Simoa platform^23,24^. CV%s of the assays were 8–29%, which shows the need to improve the analytical performance of the assays. However, in the clinically relevant levels of S-EGFR and S-HBEGF, the CV%s were 11% and 15%, respectively, which is comparable to commonly used biomarker assays. Furthermore, standardized cut-offs for EGFR and EGFR ligands have not yet been established, so in the current study we estimated cutoffs using Youden’s method for optimal cutoffs. Finally, the 5-year OS for the entire study population of early-stage breast cancer patients was 90%, whereas the 5-year OS for early-stage breast cancer patients in Denmark in a national database was 77% in the period 2005–2009^32^. Thus, 5-year OS rate is higher in the present study population as compared to the national population of early-stage breast cancer patients. However, when considering specific age-groups, the 5-year OS are overall comparable between age-groups and the difference in 5-year OS might, thus, reflect differences in age distribution between the present study population and the nationwide study population^25,32^. For instance, in the age-category 65–74 years the 5-year OS is 80% in both the study population and nationwide population^32^.

The study was conducted in accordance with REMARK^21^ and used outcome measures as recommended in the Proposal for Standardized Definitions for Efficacy End Points in Adjuvant Breast Cancer Trials: The STEEP system^26^, thus enabling researchers to assess and compare results in future studies.

In conclusion, the present study demonstrated significantly shorter survival in early-stage breast cancer patients with low pretreatment levels of either S-EGFR (< 60.3 ng/mL) or S-HBEGF (< 21.4 pg/mL). Thus, the findings indicate shorter survival in patients with low S-EGFR not only in metastatic breast cancer, as shown in previous studies, but also in early-stage breast cancer. The prognostic value of S-HBEGF in breast cancer patients has not previously been investigated. The findings indicate that in subgroups of breast cancer patients, the EGFR-pathway is more involved in the malignant potential than in others. The current study results need validation in well-defined independent study populations including subgroup populations defined as hormone receptor positive patients, HER2-positive patients and triple negative patients. Furthermore, it would be of interest to investigate predictive value of these biomarkers in populations that include breast cancer patients receiving EGFR-co-targeted treatments. Overall, the results of this study indicate a prognostic value of S-EGFR and S-HBEGF in early-stage breast cancer and could be a stepping stone for further investigation of S-EGFR and S-HBEGF as prognostic biomarkers in breast cancer. Furthermore, it would be of interest to investigate the predictive value of EGFR and EGFR ligands, which is undetermined in breast cancer.

## Supplementary information

Supplementary information.

## Data Availability

The dataset contains person-sensitive data that were used under license for the study. Thus, the data are not publicly available. Upon reasonable request and with permission from the relevant legal authorities under existing laws, the data may be made available by the authors.
