# Supplementary material for: Prognostic impact of serum levels of EGFR and EGFR ligands in early-stage breast cancer
Source: Sci Rep. 2020 Oct 6;10:16558. doi: 10.1038/s41598-020-72944-1 (PMC7538553; doi:10.1038/s41598-020-72944-1)

Supplementary information

Prognostic impact of serum levels of EGFR and EGFR ligands in early-stage breast cancer

Ina Mathilde Kjær*[1,2], Dorte Aalund Olsen[1], Ivan Brandslund[2], Troels Bechmann[2,3], Erik Hugger Jakobsen[3], Søren Bie Bogh[4], Jonna Skov Madsen[1,2]

[1]Department of Biochemistry and Immunology, Lillebaelt Hospital, University Hospital of Southern Denmark, Denmark

[2]Department of Regional Health Research, Faculty of Health Sciences, University of Southern Denmark, Denmark

[3]Department of Oncology, Lillebaelt Hospital, University Hospital of Southern Denmark, Denmark

[4]OPEN, Open Patient data Explorative Network, Department of Clinical Research, University of Southern Denmark, Denmark

*Corresponding author: ina.mathilde.kjaer@rsyd.dk

Appendix 1 - Pre- to postoperative changes in S-EGFR (a), S-EGF (b), S-HBEGF (c), S-AREG (d), S-TGFα (e) and S-BTC (f) in 113 early-stage breast cancer patients by 5-year overall survival. The preoperative sample was obtained before primary surgery for breast cancer and the postoperative sample between 14 and 30 days after surgery.

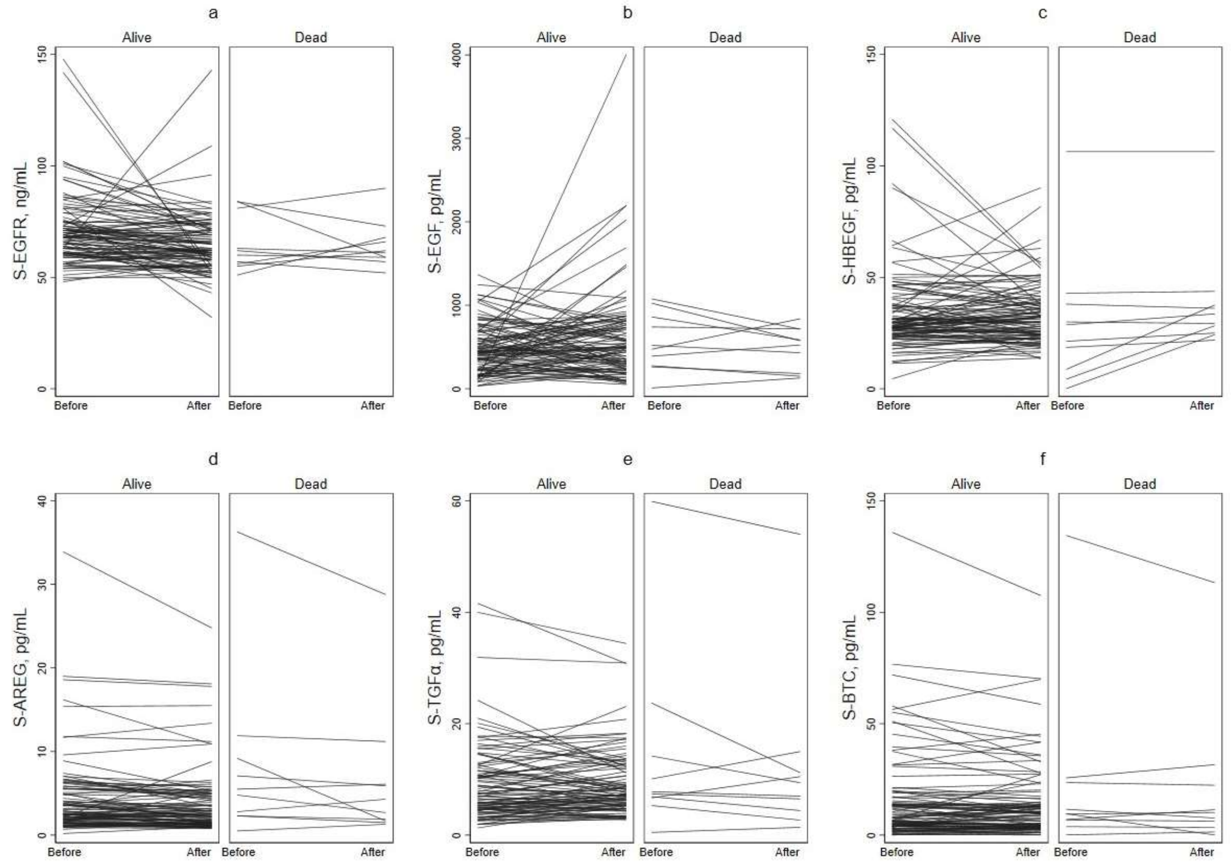

Alive: Alive five years after primary breast cancer surgery; Dead: Dead within five years after breast cancer surgery; Before: Preoperative serum sample; After: Postoperative serum sample obtained within 14–30 days after surgery.

Appendix 2 - Pre- to postoperative delta-values of S-EGFR (a), S-EGF (b), S-HBEGF (c), S-AREG (d), S-TGFα (e) and S-BTC (f) in 113 early-stage breast cancer patients by 5-year overall survival. The preoperative sample was obtained before primary surgery for breast cancer and the postoperative sample between 14 and 30 days after surgery.

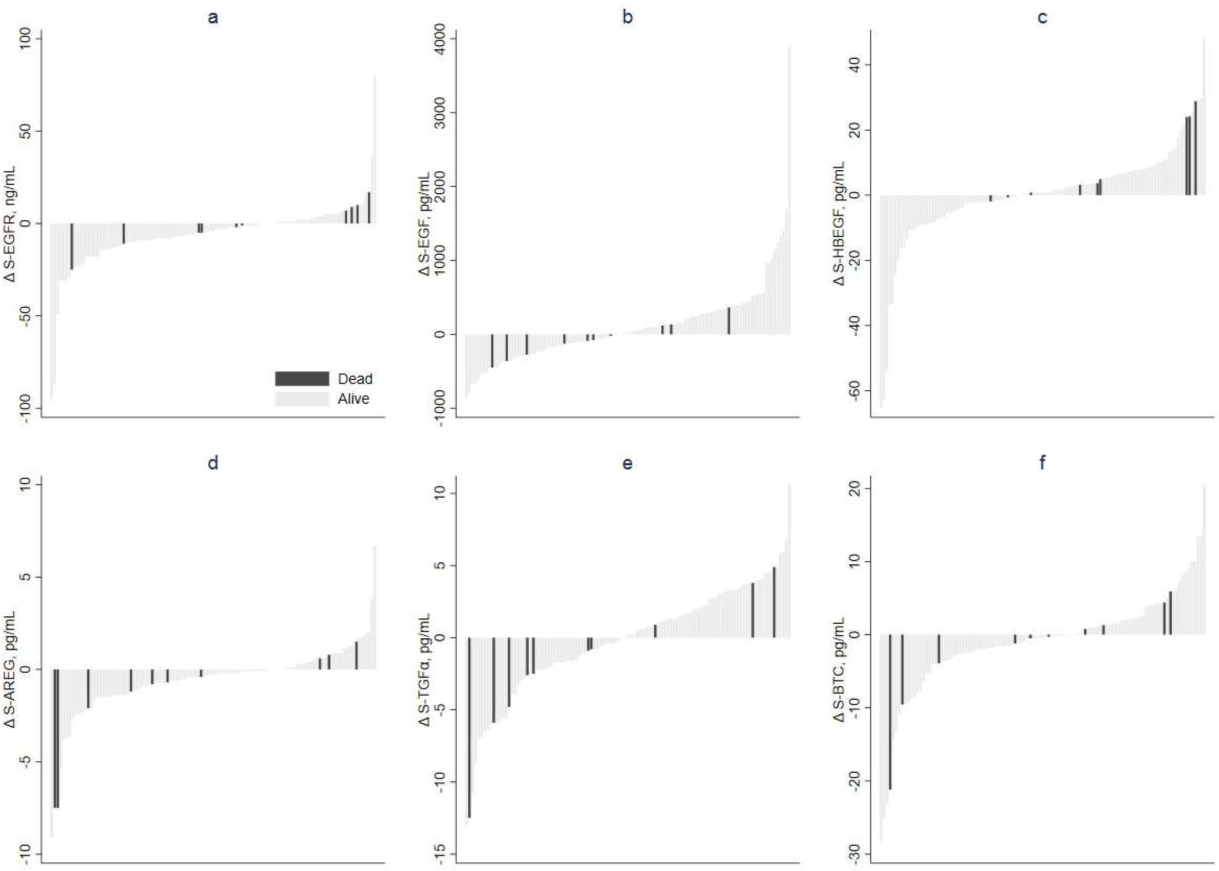

Alive: Alive five years after primary breast cancer surgery; Dead: Dead within five years after breast cancer surgery

Appendix 3 - Changes in S-EGFR (a), S-EGF (b), S-HBEGF (c), S-AREG (d), S-TGFα (e) and S-BTC (f) in 14 breast cancer patients. The preoperative sample was obtained before primary surgery for early-stage breast cancer (n=14). The postoperative sample was obtained between 14 and 30 days post-surgery (n=12). The recurrence sample were obtained within three months before systemic recurrence of breast cancer (n=14). For HB-EGF only 12 patients had enough serum for analysis of the preoperative sample.

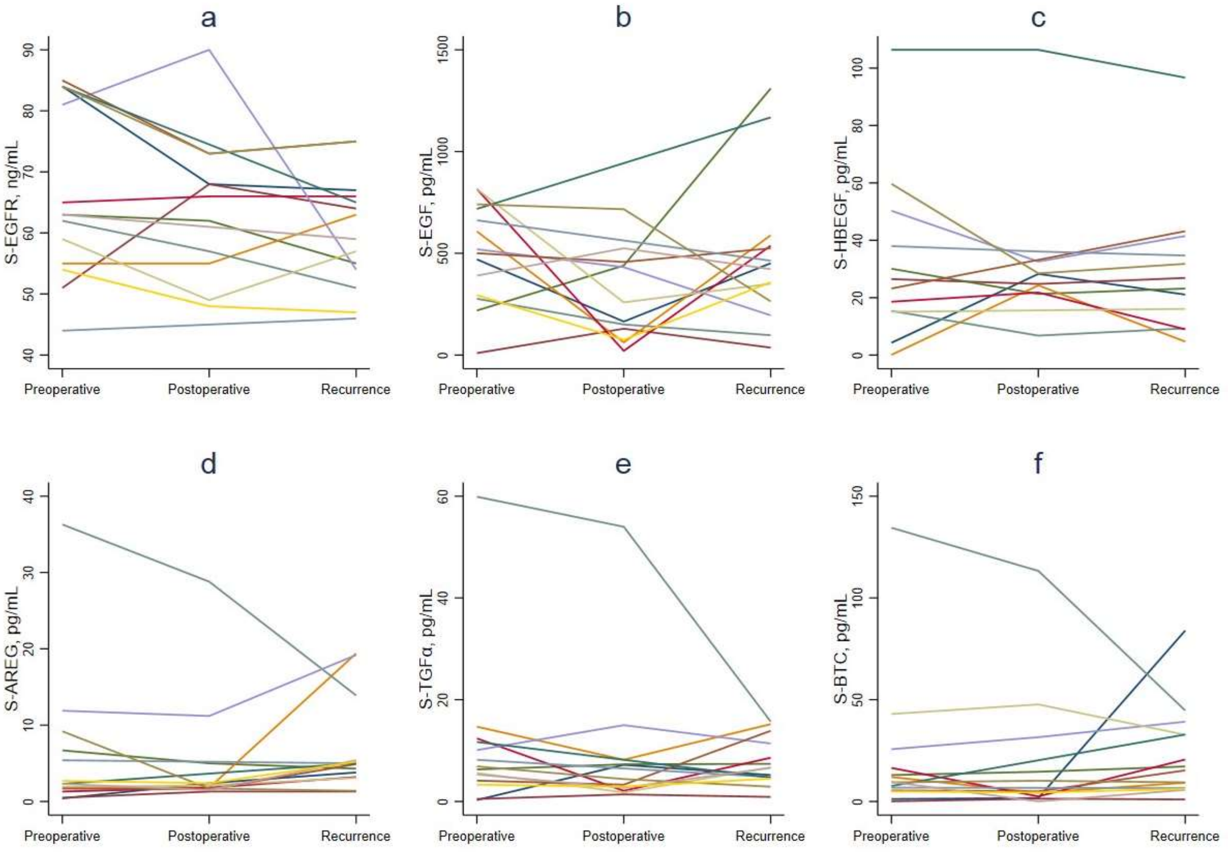

Supplement: Supplementary file 1 — Supplementary information. [file 41598_2020_72944_MOESM1_ESM.pdf]
